# Newspaper Attack on the Code of Ethics

**Published:** 1897-06

**Authors:** 


### NEWSPAPER ATTACK ON THE CODE OF ETHICS.

Barring the fact that the daily newspapers print anything that pays in the way of quack advertisements, not excluding those that are unblushingly fraudulent or immoral, physicians have no reason, as a rule, to complain of the treatment accorded them by the lay press. In fact, even the offense just mentioned is injurious a thonsand-fold more to the public than to the medical profession. The New Orleans papers have been particularly just to the doctors, hence it is with regret that we feel in honor bound to protest against an unfair attack upon the Code of Medical Ethics which appeared last month in the columns of the *Times-Democrat*, of this city. An account of a meeting of the Atlanta Society of Medicine, at which charges were preferred against some members for a supposed violation of the code, is introduced as follows:

"It is questionable whether there has ever been such a disturbance among the medical practitioners of a Southern city as there is now and has been a week among the medical men at Atlanta, Ga. The disturbance, it would be almost unnecessary to say, is in connection with that moss-grown fossil, the Medical Code of Ethics."

After a sensational account of the meeting, taken mainly from the Atlanta *Constitution*, the editorial closes with this hit not only at the code, but chiefly at the large majority of the regular profession:

"The supporters of that curio, the Code, are mostly physicians and surgeons who got themselves amply advertised through hospitals, colleges, etc., with which they are connected, and having these channels of advertisement themselves, they want none of their medical brethren to be otherwise advertised, which is a paying arrangement for them, but not for the others."

We find an answer so ready to hand that we appropriate it without hesitation. It forms part of the report of the Board of Censors of the Atlanta Society of Medicine on this very incident. They first acquit the doctors of the charge against them (of having published an account of a surgical operation in a daily dewspaper), as it was clearly proved by testimony under oath that the three physicians accused had nothing to do with the publication. They continue:

"In view of the fact that recent publications in the daily press of this city have given great notoriety to the proceedings of the Atlanta Society of Medicine, and further that the 'Code of Medical Ethics' which this society professed to maintain has been grossly misrepresented and perverted by such sensational articles to the manifest impairment of the dignity and influence of our honored profession, your committee respectfully recommends that this report be published in the interests of all concerned. A wise man has well said that 'there is nothing so dangerous as ignorance,' therefore, for the enlightenment of those ignorant of the true spirit and intent of the 'Code of Medical Ethics,' we would state that only the grossest ignorance or most malicious perversion of the truth could possibly distort this wise, beneficent, and moral instrument into 'an antiquated engine of torture,' 'a relic of the dark and barbarious ages,' 'dragged forth' by 'a set of jealous and rusty old fogies' for the 'inquisitorial' purpose of 'crushing out the progressive spirit, rising genius' and 'advanced views' of 'enlightened doctors in their skillful efforts to relieve suffer-humanity.' The medical profession, especially as organized in its various societies, like every other organization, be it a church, a court of law, or any other civic body, has its laws, its regulations, and methods of procedure. The common experience of mankind has shown the imperative necessity of such laws and regulations, and for such standards of conduct or 'ethics,' where the beneficent and far-reaching results of united and harmonious organization are sought. The medical profession is no exception to this law. It would indeed be strange if it were otherwise, for surely there is no profession or calling in which the interests of society in general are more vitally and delicately involved! Hence it is we find that the medical profession has a 'code' of laws or 'ethics' governing its members in their varied duties and relations to the public and to themselves. This 'code,' as we now have it, is the result of the experience and practical test of many centuries. It is true that our present 'code,' which is that of the American Medical Association, is but little more than forty years of age, yet looking backward we find that its essential principles were inculcated in the famous 'oath of Hippocrates,' the so-called 'father of medicine,' and was administered to members entering our profession some 357 years before Christ. Our benevolent and noble calling in its history indeed extends so far back into the past that its history, in the proper sense of that term, is lost in traditions amidst the shadows and glooms of a remote antiquity. * * * * *

Emerging from darkness, it has seen the dawn of a better day, and has endeavored by its principles incorporated in its 'Code of Ethics' to illustrate the teachings of the 'Great Physician:' 'Whatsoever ye would that men should do to you, do ye even so to them.' In enforcing its mandates and demanding allegiance to its principles this 'code' does but claim 'that which is lawful and right.' Liberal and true, the 'Code of Medical Ethics' has been established by this profession, which has been compelled to recognize that as in the case of the church, yea! even of the 'twelve' who followed the Christ, there are those unworthy of their 'high calling.' From earliest times there have been those who, under the name of physicians, prey upon the necessity and credulity of mankind. Our 'code,' so far from 'repressing discovery' or 'retarding' the perfecting of its art, encourages and even demands that each of its members shall contribute of his 'talents,' be it 'one' or 'five,' to the advancement of all, and that the good of mankind. Through our medical societies and medical periodicals such discoveries and improvements are made known, are tested, and accepted or rejected, and that after careful and wise investigation; they are not paraded to an uninstructed and credulous public, through the medium of paid or other advertisements in the secular press.

"'Beneficent in design, kindly in its guiding hand, true in sentiment and noble in practice! !' Such is the 'genius' of our 'code,' standing pure and uncontaminated amidst certain influences of this age, which tend to lower our standard of professional honor."

Reinforcing the above, and as a retort to slurs against the "code," by some who probably have never read it through attentively, we can quote a paragraph from the address of Prof. S. E. Chaille to the medical graduates of Tulane of 1897, recently published in the JOURNAL: "You will find inclosed in the diploma of every one of you a copy of the Code of Ethics of the American Medical Association. This code commands the allegiance of the most reputable members of the medical profession; it admonishes the physician of his obligations to the public, to his patients, to his fellow physicians and to himself; it maintains in all things the honor and dignity of the medical profession, and it upholds a standard emblazoned with the sacred command: 'Do unto others as you would be done by.' If you are to be esteemed as worthy members of the medical profession, if you are to be cherished as beloved sons of this college, you will subscribe to this code and honor it in your careers, as the soldier honors the flag waving at the front of his command."—*New Orleans Medical and Surgical Journal.*

